# A survey of visitors on Swedish livestock farms with reference to the spread of animal diseases

**DOI:** 10.1186/1746-6148-9-184

**Published:** 2013-09-16

**Authors:** Maria Nöremark, Jenny Frössling, Susanna Sternberg Lewerin

**Affiliations:** 1 Department of Disease Control and Epidemiology, SVA, National Veterinary Institute, 751 89 Uppsala, Sweden; 2 Department of Animal Environment and Health, SLU, Swedish University of Agricultural Sciences, Box 234, SE-532 23 Skara, Sweden; 3 Department of Biomedical Sciences and Veterinary Public Health, SLU, Swedish University of Agricultural Sciences, Box 7028, 750 05 Uppsala, Sweden

## Abstract

**Background:**

In addition to livestock movements, other between-farm contacts such as visitors may contribute to the spread of contagious animal diseases. Knowledge about such contacts is essential for contingency planning. Preventive measures, risk-based surveillance and contact tracing may be facilitated if the frequency and type of between-farm contacts can be assessed for different types of farms. The aim of this study was to investigate the frequency and types of visitors on farms with cloven-hoofed animals in Sweden and to analyse whether there were differences in the number of visitors attributable to region, season, and type of herd. Data were collected from Swedish farmers through contact-logs covering two-week periods during four different seasons.

**Results:**

In total, 482 (32%) farmers filled in the contact log for at least one period and the data represent 18,416 days. The average number of professional and non-professional visitors per day was 0.3 and 0.8, respectively. Whereas the number of professional visitors seemed to increase with increasing herd size, this relation was not seen for non-professional visits. The mean numbers of visitors per day were highest in the summer and in the farm category ‘small mixed farm’. Reports of the visitors’ degree of contact with the animals showed that veterinarians, AI-technicians, animal transporters and neighbours were often in direct contact with the animals or entered the stables and 8.8% of the repairmen were also in direct contact with animals, which was unexpected. In a multivariable analysis, species, herd size and season were significantly associated with the number of professional visitors as well as the number of visitors in direct contact with the animals.

**Conclusion:**

In conclusion there was a large variation between farms in the number and type of contacts. The number of visitors that may be more likely to spread diseases between farms was associated with animal species and herd size.

## Background

Contagious livestock diseases have a negative impact on production and farm economy as well as animal welfare. Moreover, several of the diseases in livestock are zoonotic and affect human health. There are thus major reasons to prevent and control these diseases, both endemic and exotic diseases. In infectious disease prevention and control, an important key is to understand the contact patterns and thus potential routes of spread between livestock farms within a country. Because one of the most important routes of spread is direct contact between live animals, livestock movements are often registered in central databases [1]. However, many diseases, e.g. foot- and mouth disease, classical swine fever, bovine viral diarrhoea and Aujezsky’s disease, can also spread via indirect contacts, such as farm visitors, transports or shared equipment [2]. In contrast to data on livestock trade, these indirect contacts are seldom registered centrally.

Assessments of the type and frequency of contacts such as visitors can be used in contingency planning as an indication of what can be expected regarding number of contacts during an outbreak. This information can be relevant for assessing potential spread and when designing forms for contact tracing. The identification of farm characteristics associated with more frequent contacts can also be useful input for prioritizations in contact-tracing and in the design of risk-based surveillance activities.

The risk of disease spread via visitors can be minimized by preventive biosecurity measures such as use of clean protective clothing and boots (preferably provided by the farmer), cleaning of equipment used on the farm and hand wash [3-6]. However, from a previous study in Sweden it is clear that farmers perceive the risk of disease introduction as low and are not always motivated to apply biosecurity routines [7]. It has also been shown that there was large variation in the biosecurity routines applied by different types of professional visitors [7]. In order to increase awareness among farmers and veterinarians, as well as other visitors, specific information campaigns related to disease prevention and control can be performed. In such activities, knowledge about the average number and type of visitors in different types of farms can be very useful, as it enables targeting of high risk farm categories and visitors. This knowledge is also important when risks for disease spread through indirect contacts and possible contact patterns are communicated. Furthermore, the expected number of contacts is often needed as input data in mathematical modelling of disease outbreaks and for the highly contagious diseases the indirect contacts are also relevant [8,9]. Such modelling can in turn be used to approximate the extent of an outbreak and to assess possible effects of different disease control interventions.

The aim of this study was to investigate the frequency and types of visitors as potential indirect contacts between farms with cloven-hoofed animals in Sweden and to analyse whether there were differences in the number of visitors attributable to region, season, and farm characteristics such as herd size or species present on the farm.

## Methods

### Selection of farms and contact log

This study was based on data collected through a mailed contact log that was sent to Swedish livestock farmers in 2006 and 2007. The participants were asked to register visitors and other farm contacts daily during four two-week periods, throughout the different seasons of the year. In total, the contact log was sent out on five occasions, covering all four seasons, i.e. July 2006, November 2006, February 2007, April 2007, and July 2007. The reason for the fifth round sent out in summer 2007 was to ensure that data was obtained across seasons also for these farmers who joined the study in the 2nd round.

The data collection was done in parallel with a questionnaire dealing with on-farm biosecurity routines [7], i.e. farmers were asked to respond to both the biosecurity questionnaire and to document contacts in the contact logs. Data from the biosecurity questionnaire regarding animal species present on the farm and herd size were also used in this study. The selection process is described in detail in the cited paper. In summary, a stratified random sample of farmers was selected in five different regions, from the very south to the north of Sweden to capture different geographical density and different predominant production. From each region, approximately 200 cattle farmers and 120 pig farmers with different production systems were selected, as well as 40 sheep farmers and 20 goat farmers (not all regions had this many farmers), resulting in a total of 1498 farmers. The basis for the sample was the official register of animal holdings at the Swedish Board of Agriculture. The sample size was a compromise between (i) having enough data to analyse, (ii) expected return rate, (iii) time limitations for data entry, and (iv) number of holdings in the regions.

The contact log forms were sent by mail approximately ten days before the start of the period of data collection. Each time an accompanying letter was enclosed, in the first round it described the background of the study and on consecutive occasions it reminded participants of the purpose of the study and encouraged continued participation. Farmers were informed that their replies would be treated anonymously and for each round an instruction on how to fill in the log was included. A response envelope (free of charge) was included and a lottery ticket was enclosed as a sign of gratitude. The study was prospective and participants were asked to record data on a daily basis for the defined period. To avoid retrospective data collection and potential recall bias reminders were therefore not sent. However, unless farmers declined participation, we continued to send contact log forms for the remaining periods if they had responded to at least one previous period.

The contact log forms were prospective and designed as a table of the different types of contacts and with one page per day (an English translation of the contact log is available as e-supplementary Additional file 1). The different types of contacts specified were transports, professional visitors, other visitors, livestock, dead-stock, shared equipment and farmers’ own visits to other farms. Farmers were asked to indicate the number of visitors of each type and their level of contact, i.e. if the visitor was in direct contact with the livestock, entered the stable or stayed outside the stable, and if they were livestock owners. Before submission to the participants, the contact log was tested on a reference group of veterinarians specialised in disease control and thereafter on six farmers.

### Background population

The animal species of interest in this study were cattle, pigs, sheep and goats. In 2006, there were approximately 25,000 agricultural enterprises with cattle in Sweden and of these, 8027 had cattle for milk production [10]. The average cattle herd size was 64 cattle (or 48 dairy cows, 14 suckler cows). Furthermore, there were 2,414 companies with pigs. The average pig herd size was 116 sows and 495 piglets and pigs for fattening. Moreover, there were 9,152 agricultural enterprises with sheep and of these, one third had <10 ewes and only 14% had >50 ewes [10]. In total, there were approximately 5,500 goats in the whole country. However, information on the number of holdings with goats was not available in the official statistics. For all species, the population is concentrated to the southern parts of the country. Since 2006, the number of pig herds and dairy cattle herds has decreased and the average size of herds has increased [11].

### Data management and editing

The contact logs were entered into a Microsoft Office Access database by single entry. In the editing of the data, some assumptions were made. In the instructions farmers were asked to use integers when registering the number of contacts and when editing the data “x” or “yes” were constantly interpreted as “1”, unless other information indicated that another integer should be used. Furthermore, the data were scrutinised after entry and whenever there were indications of typing errors, data were checked and corrected. For herds where data were available for two summer periods, the first was kept in the dataset while the second was dropped.

The parallel questionnaire on biosecurity routines [7] included questions on herd size and species present on the farm, and these data were also used in this study. The categories of species were cattle, swine, sheep or goats, and mixed. A herd was considered mixed if animals of more than one of the other categories were present. For herd size, three classes were created; hobby, medium and large. The aim of the classification was to create groups reflecting different levels of production intensity. This was based on the number of animals on the holding reported in the questionnaire, and the limits were set using the rather rough assumptions that hobby farmers do not earn their living from their livestock production and that large farms will in general need employed staff were used. For cattle and pig farmers, herd sizes <15 cattle and <20 pigs, respectively (corresponding to farms below the 30^th^ percentile) were classified as hobby and >150 cattle and >1500 pigs, respectively as large (above the 90^th^ percentile). For sheep and goats, <50 animals were classified as hobby (below the 85^th^ percentile), and >300 animals as large (above the 98.5^th^ percentile).

Locations of the herd were denoted according to the Nomenclature of Territorial Units for Statistics level 2 which divides Sweden into eight regions [12]. Five of these regions were represented in the study; Övre Norrland, Östra Mellansverige, Småland med öarna, Sydsverige and Västsverige. In the statistical analysis, visitors were categorised into either professional or non-professional: veterinarians, AI technicians, inspectors, transporters, hoof trimmers, repairmen etc. were considered professional visitors, while e.g. visitors on field trips, neighbours and customers in farm shops or Bed & Breakfast enterprises were considered non-professional visitors.

### Data analysis

Descriptive statistics were obtained for the different types of visitors and levels of contact, by species category, herd size, region and season. Furthermore, the proportion of visitors reported to have livestock of their own was calculated.

Considering their expected high influence on the risk of disease spread, special attention was given to the number of professional visitors and the number of visitors with direct contacts with the animals. These two outcomes were further investigated using regression models where possible associations between number of visitors per two-week period and different explanatory variables were analysed. The potential explanatory variables investigated were; species, herd size, region and season. Associations between outcomes and explanatory variables were first investigated by univariable regression. The outcome variables also contained an excess of zeroes and zero-inflated negative binomial regression was therefore chosen. In this type of model, a binary (here logistic) model and a negative binomial model are fit simultaneously to capture both the probability of zero counts and the probability of non-zero counts [13]. Because farmers contributed with several observations (i.e. one observation per season), robust standard errors were applied with clustering on herd level. Potential variables were tested in both the logistic and the negative binomial parts of the model in a stepwise process using backward elimination. The limit for keeping the variable in the model was set to p < 0.10. Biologically relevant interactions between the remaining variables were tested and interaction terms were kept if significant at the 0.05 level. The fit of the final models was examined by comparing the observed and predicted values for the different covariate patterns.

### Software used

Data was entered and stored in Microsoft Office Access 2007 (Microsoft Co., Redmond, Washington, USA), and analysed using Stata Statistical Software: Release 11.2 (StataCorp. 2009, College Station, Texas, USA).

## Results

### Response rate

Out of the selected farmers, 482 (32%) responded to the contact log on at least one occasion. The numbers of responses per period were as follows: summer ‘06 n = 427, autumn ‘06 n = 235, winter ‘06 n = 289, spring ‘07 n = 327 and summer ‘07 n = 241. The number of farmers that sent in one, two, three, four or five contact logs was 85, 76, 93, 137 and 95 respectively. After data cleaning, the responses represent a total of approximately 1,315 two-week periods (18,416 days). The number of responding farmers and response rate by different categories of registered species on the holding and by region is shown in Table 1. Reasons for non-response were given by 21% of non-responders [7]. The most important reason for non-response among these farmers was “ceased animal production” (50%). Notably, one reason given by a few farmers was “having too many visits to keep track of”.

**Table 1:** Response rate

	**Sample**	**Responders**	
	**N**	**n**	**%**
Response-rate, by:			
*Registered species on the holding:*			
Cattle	640	178	26.2
Pigs	589	190	28.4
Sheep/goats	269	101	37.5
Missing*		13	
Total	1498	482	32.2
*Region (NUTS level 2):*			
Övre Norrland	293	71	24.2
Östra Mellansverige	258	82	31.8
Småland med öarna	270	93	34.4
Sydsverige	362	115	31.8
Västsverige	308	107	34.7
Other	7	14	
Total	1498		

Contact-log response rates disaggregated by farm types according to species and by regions.

*Selection was based on species and region. Reported species was not always consistent with species registered on farm. These respondents had not used the coded response letter and their selection strata could therefore not be identified.

### Descriptive statistics

**Figure 1:**
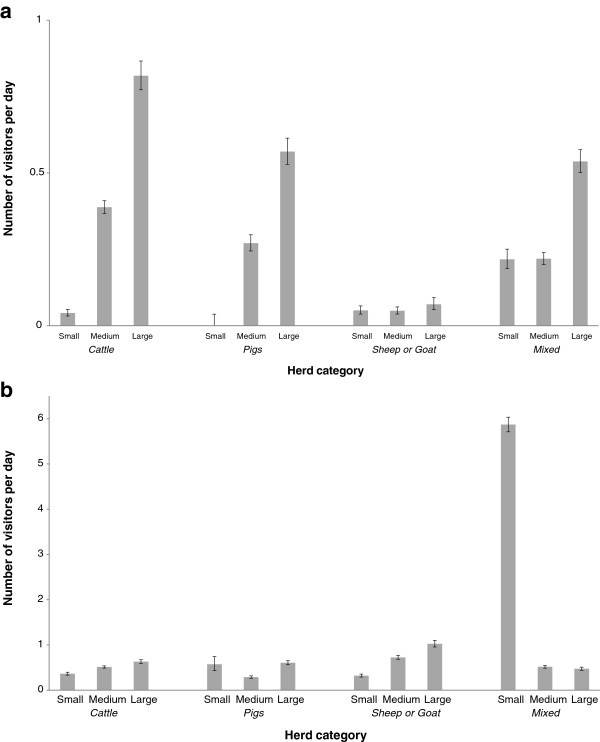
**a-b Number of visitors in different categories of herds.** Results from a study dealing with frequency of indirect farm-to-farm contacts (Swedish livestock farms, 2006–2007). The bars represent the mean number of **a)** professional visitors, and **b)** non-professional visitors, by categories of species and herd size, black lines show the 95% confidence interval.

According to the replies, 45 herds (9.3%) had no visitors at all during any of the two-week periods. There were 111 herds (23.0%) that did not have any professional visitors and 133 herds (27.6%) that did not have any non-professional visitors. On average, the number of visitors per day was 1.1 (range 0–221). The mean number of professional visitors was 0.3 (median 0; range 0–18) and the mean number of non-professional visitors per day was 0.8 (median 0; range 0–221). The numbers of professional visitors, non-professional visitors and visitors in direct or indirect contact with animals are given by category of species and herd size in Figure 1a-b.

**Figure 2:**
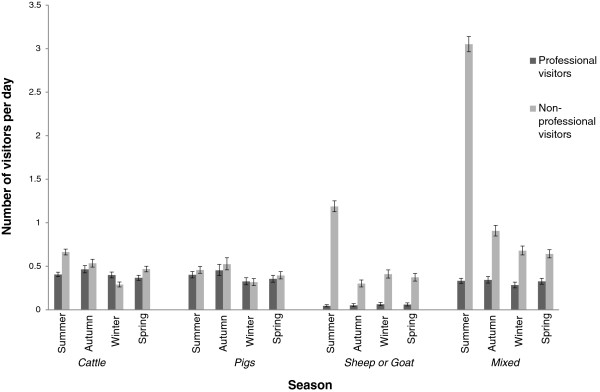
**Number of visitors by season, category of species and type of visitors.** Results from a study dealing with frequency of indirect farm-to-farm contacts (Swedish livestock farms, 2006–2007). The graph shows the mean number of visitors per day, by category of species and season. The dark grey bars refer to professional visitors, light grey bars refer to non-professional visitors and the black lines show the 95% confidence interval.

The descriptive statistics indicated differences between daily mean numbers of visitors related to herd size (Figure 1a-b). Whereas the number of professional visitors seemed to increase with herd size, this relation was not seen for non-professional visits. Moreover, there were differences related to species present on the farm. For example, veterinary visits in cattle herds seemed to increase with herd size but this tendency was not obvious for pig herds or for sheep herds. The highest mean number (6.5) of non-professional visitors per day was found in the category ‘small mixed farm’. When seasons were compared, the average number of visitors was higher in summer. This difference was most obvious as regards non-professional visitors in mixed herds and herds with sheep or goat (Figure 2).

Farmers registered information on the animal ownership of the non-professional visitor for 88% of reported visits (3696 occasions). However, they often indicated this with an “x” or “yes” instead of the actual number of visitors having livestock and these data were therefore analysed on occasion and type of visitor. The results are shown in Table 2. The largest proportion of visitors reported to own livestock was found in the neighbour category.

**Table 2:** Visitors’ animal ownership

			**Visitors' livestock ownership (n is numer of occasions, and percentage is in relation to number of occasions when ownersip was reported)**											
**Type of visitor**	**Reported occasions of visit**^**a**^**(total)**	**Occasion of visits where information on visiors' livestock ownership was reported**	**Cattle**		**Pigs**		**Sheep**		**Goats**		**No livestock**		**Unknown to reporting farmer**	
			**n**	**%**	**n**	**%**	**n**	**%**	**n**	**%**	**n**	**%**	**n**	**%**
Field trip	103	68	12	17.6	2	2.9	1	1.5	1	1.5	37	54.4	16	23.5
Bed & breakfast guest	60	60	2	3.3	0	0.0	0	0.0	0	0.0	47	78.3	11	18.3
Customer in farm shop	252	212	14	6.6	2	0.9	19	9.0	9	4.2	35	16.5	145	68.4
Neighbour	1413	1268	413	32.6	102	8.0	131	10.3	3	0.2	772	60.9	19	1.5
Other non-professional	2371	2088	185	8.9	66	3.2	80	3.8	14	0.7	1629	78.0	258	12.4

^a^The numbers represent occasions of visits and not the number of visitors due to the way data was reported. One occasion can represent one or more visitors.

Reported livestock ownership for non-professionals visiting Swedish livestock farms. Data represent 1,315 two-week periods (18,416 days) recorded by 482 farmers.

The locations within the farm where visitors were reported to enter are shown in Table 3. As expected, veterinarians and AI-technicians were often in direct contact with animals. Animal transporters were also often in direct contact with the animals (39.3%). Among dead-stock collectors, on the other hand, few were in contact with animals (6.1%) or entered the stables (5.1%). Notably, 8.8% of the repairmen were in direct contact with animals and neighbours were in direct contact or entered the stables at 36.5% of their visits.

**Table 3:** Visitors’ level of contact with animals

**Type of visitor**	**Type of contact**						**Total number**
	**Direct contact**		**In stable**		**On farm**		
	***n***	***%***	***n***	***%***	***n***	***%***	
*Professional visitors*							
Milk truck (driver)	3	0.2	3	0.2	1592	99.6	1598
Temporary employee	502	80.4	58	9.3	64	10.3	624
Animal transporter (live or for slaughter)	197	40.6	112	23.1	176	36.3	485
Feed truck (driver)	0	0.0	8	2.3	341	97.7	349
AI-technician	301	88.0	20	5.8	21	6.1	342
Other type of professional visit	82	27.6	72	24.2	143	48.1	297
Repairman	25	8.8	97	34.0	163	57.2	285
Veterinarian	240	85.4	29	10.3	12	4.3	281
Transporter of dead stock	4	4.7	4	4.7	78	90.7	86
Salesman	1	1.2	18	22.2	62	76.5	81
Production advisor	17	34.0	8	16.0	25	50.0	50
Hoof trimmer	32	97.0	0	0.0	1	3.0	33
Inspector from municipality or county	8	29.6	11	40.7	8	29.6	27
Sheep shearer	15	93.8	0	0.0	1	6.3	16
Sample collector (control programme)	7	58.3	4	33.3	1	8.3	12
*Non-professional visitors*							
Other visits	151	12.1	318	25.4	781	62.5	1250
Neighbours	103	15.4	157	23.4	411	61.3	671
Customers in farm shop	2	2.4	4	4.9	76	92.7	82
Field trip	21	37.5	22	39.3	13	23.2	56
“Stay on a farm”	7	24.1	1	3.4	21	72.4	29

Level of contact with the animals at the farm among different categories of visitors, based on 1,315 two-week periods (18,416 days) recorded by 482 Swedish livestock farmers in 2006–2007. The numbers presented in the table are based on visits where type of contact was specified.

### Results from multivariable regressions

In the final models, the number of professional visitors, as well as the number of visitors in direct contact with animals were significantly associated with species, herd size and season (Tables 4 and 5). For both of the outcomes, species and herd size were included in the negative binomial part of the model (representing counts of visitors) while species, herd size and season were included in the logistic part of the model (representing the probability of no visits at all). Although an interaction between herd size and species was found, the models were not stable with this interaction term (i.e. resulted in extreme incidence risk ratios and confidence intervals) and it was therefore excluded from the final models. The geographical differences seen in the univariable analyses were not observed when other risk factors were accounted for. For both professional visitors and visitors in direct contact with animals, visits were more likely in large herds compared to small and medium herds. From herds reporting these types of visits, there were also more visitors in herds with cattle, compared to other species. However, the numbers of visitors in direct contact (i.e. including both professional and non-professional) were higher in hobby farms compared to large and medium sized farms. In addition, professional visits were less likely in summer compared to spring and autumn.

**Table 4:** Regression results, number of professional visitors

**Variable**	**Coef.**	**95% confidence interval**		***P***
**category**				
*Negative binomial part of the model*				
Constant	1.87	1.02	2.72	<0.001
Animal species				
cattle				
swine	−0.48	−0.75	−0.22	<0.001
sheep or goat	−2.15	−2.78	−1.52	<0.001
mixed	−0.33	−0.61	−0.05	0.023
Herd size				
hobby				
medium	0.01	−0.86	0.89	0.974
large	0.59	−0.28	1.47	0.185
*Logistic part of model (probability of no visitors)*				
Constant	1.08	0.30	1.85	0.007
Animal species				
cattle				
swine	−2.36	−4.73	0.01	0.051
sheep or goat	−1.33	−5.00	2.33	0.476
mixed	0.05	−0.54	0.64	0.867
Contact log period				
summer				
autumn	−0.89	−1.60	−0.18	0.014
winter	−0.39	−0.92	0.14	0.149
spring	−0.52	−1.00	−0.05	0.031
Herd size				
hobby				
medium	−1.80	−2.88	−0.72	0.001
large	−3.49	−5.14	−1.84	<0.001

Estimates from a negative binomial regression model used to analyse the number of professional visitors in Swedish livestock herds, based on replies to a contact log sent out in 2006–2007.

**Table 5:** Regression results, number of visitors in direct contact with animals

**Variable**	**Coef.**	**95% confidence interval**		***P***
**category**				
*Negative binomial part of the model*				
Constant	3.03	1.86	4.21	<0.001
Animal species				
cattle	baseline			
swine	−0.53	−0.92	−0.13	0.009
sheep or goat	−1.47	−2.31	−0.63	0.001
mixed	−0.46	−0.85	−0.07	0.021
Herd size				
hobby	baseline			
medium	−1.90	−3.13	−0.67	0.002
large	−1.57	−2.77	−0.37	0.010
*Logistic part of model (probability of no visitors)*				
Constant	1.73	1.11	2.35	<0.001
Animal species				
cattle	baseline			
swine	−0.94	−2.14	0.26	0.123
sheep or goat	0.59	−0.81	1.98	0.409
mixed	0.10	−0.65	0.85	0.789
Contact log period				
summer	baseline			
autumn	−0.88	−1.49	−0.27	0.005
winter	−0.21	−0.81	0.39	0.489
spring	0.04	−0.50	0.57	0.891
Herd size				
hobby	baseline			
medium	−1.83	−2.49	−1.17	<0.001
large	−4.21	−8.06	−0.35	0.032

Estimates from a negative binomial regression model used to analyse the number of visitors in direct contact with animals in Swedish livestock herds, based on a contact log sent out in 2006–2007.

## Discussion

In general, one of the most important routes of disease spread is considered to be live animal trade, and animal movements between Swedish herds have recently been described [14-16]. However, for some highly contagious diseases, indirect contacts are also a potential route of disease transmission. Introduction of animals from other herds can to some extent be avoided by limiting the purchases of animals and instead relying on within-farm recruitment. Professionals visiting the farm, on the other hand, can seldom be totally avoided. The non-professional visits could in theory be avoided, but benefits from having children and urban people visit farms in order to enable better understanding of agricultural production would then be lost. Studies to investigate these types of contacts have been done in other countries [17-21], however, information on indirect contacts between Swedish farms has been missing. The results presented here therefore contribute substantially to the knowledge of what can be expected when it comes to between herd contacts.

Based on the replies, there was a large difference in the number of visitors per farm, and substantial variation within categories of farms was observed. In general however, species and herd size were significantly associated with the number of professional visits and this is an expected finding. Depending on the type of production, different professionals will be needed and with many animals the frequency of the visits will increase. For example, if the herd is large, the probability of one animal in the herd needing veterinary care will increase compared to if the number of animals is low. The contact pattern observed for veterinary visits can also be explained by other underlying structures. For example, provided that they fulfil requirements of education and regularly veterinary visits, Swedish pig farmers are generally allowed to keep certain drugs (e.g. antibiotics) on their farm and perform first line treatment of individual animals themselves. This was at the time of the study not possible for cattle owners, and this is one explanation why the veterinary visits to pig farms did not increase with herd size to the same extent as visits to cattle farms. Another example of factors that can influence frequency of visitors is the production cycle on the farm, which will affect how often animal transporters collect animals on the farm.

In comparison, the non-professional visitors did not follow the same pattern as the professional ones. From a contingency planning and information perspective, one important finding was that a number of hobby farms with mixed species had large numbers of non-professional visits. From previous studies it was clear that hobby farmers often had low biosecurity [7]. This category of farmers has also been shown to be overrepresented among farmers who were unaware of an ongoing outbreak [22]. Although non-professional visitors may not be as important for disease spread as the professionals, who tend to visit one farm after the other, it is clear that low biosecurity and unawareness in combination with large number of contacts may present a high risk. Even if only a small proportion of these visitors are in contact with other farms, the actual number may be significant when the total number of visitors is high. With hundreds of visitors per week, tracing of contacts during outbreaks may also be extremely time consuming and difficult. Thus, information about preventive biosecurity measures is crucial in such farms. Not all visitors pose equal risk, and focus could be on hygiene measures related to visitors in direct contact with animals and especially if they are livestock owners. New legislation coming into force in Sweden in September 2013 establishes the famers’ responsibility for biosecurity related to farm visits [23]. As part of implementing the new rules, these results provide important information when communicating the risk of disease transmission through visitors to farmers. Further, the findings are relevant for strategies on how to come into contact with visitors in case of a disease outbreak. The study has identified that the number of people that would need to be reached can be very high and that many of them may not be part of the farming community, i.e. they will probably not be reached through the farmers’ press or information sent to farmers. From the results, the importance of asking the farmer at an early stage of contact tracing if they have many visitors e.g. due to hosting fields trips or having construction workers or repairmen at the farm, has been highlighted.

Another important finding is the proportion of visitors of different categories that was reported to be in direct contact with the animals or to enter the stable. These findings also need to be seen in light of previous findings, where use of protective clothing was examined [7]. For example, salesmen and repairmen were reported to have poor use of protective clothing, and many farmers did not require such usage, whereas this study found that that one fourth of the salesmen entered the stables and almost nine percent of the repairmen were in direct contact with animals. Many visitors within these categories of professionals may not have an education related to animal husbandry and there is a risk that they do not realise their potential role in spreading disease. These results should be considered in the design of preventive biosecurity programmes or information campaigns during disease outbreaks where it is important not to forget this category of visitors. There is a need to communicate, both to the farmer and to the visitors, the risk of disease spread through indirect contact. These results have therefore been forwarded to the Swedish Animal Health Services and the Swedish Dairy Association which are currently working on a new farm biosecurity programme.

It is often assumed that farms in the northern parts of Sweden pose a lower risk of disease introduction as they have fewer contacts. This study demonstrates that farm characteristics were more important than geographical region and that when implementing control measures region should not be considered a primary factor.

From previous outbreak investigations, it is known that it may be difficult for farmers to recall detailed information of events such as farm visits. Farmers have also been surprised when they have realised the actual number of contacts they have had. Making a single assessment at one point in time can thus lead to underestimation of the amounts of contacts. In order to avoid recall bias and underestimation of contacts, the choice was a prospective contact log. Although the aim was to make data registration as simple as possible, participation in the study was a considerable workload for the farmers. In spite of this, 32% of the invited farmers chose to participate in the study. There were farmers that only responded to one period, we did not investigate the reasons for this and can only speculate why. Some might have found the questionnaire too burdensome, and since there is a rapid structural change in Swedish agriculture with decreasing farms it is probable that some of them quit farming during the study period. There were also farmers that responded in the start and the end of the study period but missed one or two periods in the middle. Although other information would have been interesting to include in the contact log, we tried to minimise the amount of data to be collected by the farmer and did not ask about duration of contact, biosecurity routines applied during the specific visit, origin or destination of the contact. Data on distances are planned to be collected in a future study focusing on the routes travelled by professionals.

Compared to similar studies in Switzerland and New Zealand, in which farmers were also asked to register contacts during two or three week periods, the response rate in this study lies between the two (22% and 43%). However in the New Zealand study the participants were recruited through telephone calls [20,24] and the response rate was even higher (70% when non-eligible farms had been excluded) in a corresponding Dutch study where farmers were recruited by letters and telephone calls from their local veterinarians [19]. In recent European studies in UK and Belgium, data collection was based on estimates made at one point of time and farmers were not asked to register contacts continuously, and response rates are therefore not directly comparable [17,18]. As already concluded by Ribbens et al. [18], the result of these studies are not straight forward to compare either, because they focus on different groups of farmers and because the methods for data collection have differed between countries. However some findings are worth mentioning. The large variation between farms in number of contacts and association with herd size was also observed in Belgium, California, New Zealand and the United Kingdom [17,18,20,21]. In the Belgian study it was observed that professional visits more often entered the stable compared to non-professionals [18], and when removing milk trucks (which were not relevant in the Belgian study focusing on pigs) from the Swedish data the proportion of professionals entering stables was clearly higher compared to non-professionals also in Sweden. In some parts, the study from the Netherlands registered level of contact in a comparable way, and similar findings were seen with veterinarians, AI-technicians and temporary employees among the visitors most often in direct contact with animals. However, other categories differed between the two countries, e.g. animal obstetricians which occurred in the Dutch study do not exist in Sweden, and hoof-trimmers did not occur in the Dutch data [19]. This example illustrates both the constraints in comparing results from studies with different designs, and also the need for collecting country specific data.

Because half of the non-responders who explained their non-response said that they did no longer have livestock, the response rate among farmers that in fact had livestock on their farm was even higher. A few non-responders explained that their high number of contacts was the reason why they could not participate in the study. This is unfortunate because from a disease prevention perspective, farms with many contacts are of special interest. It is noteworthy that one of these farmers indicated that the farm had around thousand visits each week, due to on-farm sales. Thus, it is possible that the average number of visits reported in this study was underestimated. Another possible reason for underestimation is that farmers, in spite of the prospective study-design, forgot to fill in all visits. This was observed in a Dutch study were comparative data were available to check the registrations [19]. There was no simple way to identify differences between responders and non-responders. It can be speculated that farmers who were more interested in biosecurity and disease prevention were more likely to agree to participate in the study. If so, it is possible that the number of non-professional visits, i.e. the category of visitors that a farmer can limit, was higher than reported. However, professional contacts are needed to keep the farm running, regardless of the farmer’s attitude towards responding to questionnaires.

The results from this study reflect the large variability among farms and contribute to the understanding of the frequency and nature of indirect contacts between Swedish livestock holdings. The large number of non-professional visits in some farms, the fact that mixed-species hobby farms (potentially with low biosecurity and outbreak awareness) often had many visitors and the proportion of salesmen and repairmen entering the farm stables, are all important observations. The expected findings, such as number of visitors being related to species and herd size, are also of value as this has not been documented before in Sweden. The study results will constitute useful background information in the planning of risk-based surveillance, risk communication, biosecurity information campaigns, as well as in outbreak management and preparedness, and as input in ongoing work on modelling of disease spread where the distributions on actual numbers of contacts can be used to simulate contact patterns relevant for different types of Swedish livestock farms.

## Conclusions

There was a large variation in number of farm visitors, both professionals and non-professionals. The number of visitors that may be more likely to spread diseases between herds was associated with animal species and herd size of the farm, however the non-professional visitors did not show the same association with herd-size and there were small mixed farms with high numbers of non-professional visitors. There were expected findings with e.g. veterinarians and AI-technicians often in direct contact with animals, but also unexpected findings with e.g. more repairmen than expected being in direct contact with animals.

## Competing interests

The authors declare that they have no competing interests.

## Authors’ contributions

MN and SSL designed the study, MN collected the data and edited the database, MN and JF performed the statistical analysis and drafted the manuscript, and all authors critically revised the manuscript. All authors read and approved the final manuscript.

## Supplementary Material

Additional file 1 The contact log (in English) is available in as an additional file. Click here for file
