# Supplementary material for: A survey of visitors on Swedish livestock farms with reference to the spread of animal diseases
Source: BMC Vet Res. 2013 Sep 16;9:184. doi: 10.1186/1746-6148-9-184 (PMC3848732; doi:10.1186/1746-6148-9-184)
Supplement: Additional file 1 — The contact log (in English) is available in as an additional file. [file 1746-6148-9-184-S1.pdf]

Contacts/visits Wednesday November 15th

PPN:

Transports

Transports	Number of contacts or visits today	Number in direct contact with animals	Number in stable	Number in areas where there are no animals
Milk truck (driver)				
Feed truck (driver)				

Professional visits

Professional visits	Number of contacts or visits today	Number in direct contact with animals	Number in stable	Number in areas where there are no animals
Temporary employee				
AI-technician				
Veterinarian				
Sample collector (control programme)				
Control milking				
Hoof trimmer				
Sheep sheerer				
Production advisor				
Inspector from municipality or county				
Salesman				
Repairman				
Animal transporter (live or for slaughter)				
Transporter of dead stock				
Other type of professional visit				

☐ **No visits/contacts today**
(mark with an X if there were no contacts today)

Other visitors

Does visitor have animals?

Other visitors	Number of contacts or visits today	Number in direct contact with animals	Number in stable	Number in areas where there are no animals	cattle	pig	sheep	goat	no	do not know
Fieldtrip										
”Stay on a farm”										
Customers in farm shop										
Neighbours										
Other visits										

Animals and dead stock

Animals and dead stock	X if "yes"	
Animals entering farm (purchase or loan)		Species and number:
Animals have been to exhibition		Where:
Dead stock collected on farm		
Live animal collected on farm		
Animal for slaughter collected on farm		

Borrowed/rented/shared equipment

Received shared equipment		If yes, indicate what:
Dispatch of shared equipment		If yes, indicate what:

Visited other farm

Visited farm with cattle/pigs/sheep/goats?		If yes: which species:

Space for comments:
